# Veterinary Chiropractic Treatment as a Measure to Prevent the Occurrence of Spondylosis in Boxers

**DOI:** 10.3390/vetsci8090199

**Published:** 2021-09-17

**Authors:** Kristin Steinmoen Halle, Aksel Granhus

**Affiliations:** 1 Logreklinikken AS, Flaggstangåsen 4, 1455 Nordre Frogn, Norway; 2 Norwegian Institute of Bioeconomy Research, P.O. Box 115, 1431 Ås, Norway; aksel.granhus@nibio.no

**Keywords:** dogs, enthesophytes, spinal disease, spondylosis deformans

## Abstract

Spondylosis deformans is a spinal disease common to several dog breeds, and several treatments including veterinary chiropractic may be used to treat affected dogs. Little is known, however, about the efficacy of chiropractic treatment as precautionary measure, aiming to reduce the probability of spondylosis development. We performed a randomized study where one half of the Boxer puppies from 17 litters were given veterinary chiropractic treatment at monthly intervals from eight weeks of age until they were one year old, while the other half were given no treatment (treated: *n* = 44, controls: *n* = 43). At an age of one year, spondylosis occurrence was recorded based on a scoring of X-ray images of the spine. The frequency of occurrence was significantly lower (*p* = 0.0478) in the treated dogs (25.0%) than in the controls (46.5%). We also tested if spondylosis occurrence in the treated dogs correlated with the average number of spinal joints with decreased mobility found per chiropractic treatment. No such effect was found, however. In summary, our results suggest that veterinary chiropractic treatment may be successfully used to reduce the probability of early development of spondylosis in young Boxers.

## 1. Introduction

Spondylosis deformans, hereafter referred to as spondylosis, is a spinal disease of several vertebrates, known to occur in as widely different species such as, e.g., rats, cats, dogs, horses, and humans [1,2,3,4,5,6]. In dogs, the disease develops through a gradual calcification of the ventral longitudinal ligament, appearing on X-ray images as enthesophytes growing from the borders of the vertebrae [7,8,9,10,11,12]. Sometimes the process stops with the development of a small bone spur, whereas in other cases the calcification may develop to form a full bone bridge between the vertebrae [10,11,12]. While affected dogs might have subclinical signs like lack of motion in some segments of the spine and local mild pain [13], there are also studies that did not show any clear correlation between spondylosis and symptoms [14]. Hence, predicting what will be the consequence for the well-being of affected dogs is not straightforward.

Spondylosis is more prevalent in some breeds than in others. In an early study of spondylosis in Boxers from the USA, UK, and Sweden [8], the disease was found in about 50% of dogs by six years of age, and in 75% at an age of nine years. A similar level was found in a later study of Italian Boxers [15]. Anatomical dissection of the lumbosacral spinal columns from German Shepherds with spondylosis deformans has revealed that diseased vertebrae have more flexibility than healthy vertebrae in the sagittal and frontal planes, but less so for dorsal flexion [13]. As shown in German Shepherds, an imperfect seventh lumbar vertebra (L7) facet geometry may cause a predisposition to spondylosis, and congenital factors as well as body weight and locomotion in immature dogs may also be predisposing factors [13,16]. While examining X-ray images of the spine of Boxers, a breed with relatively high occurrence of the disease [10,17], the main author has observed a lot of very short L7 vertebrae, which could suggest that some of the same mechanisms are seen in Boxers. Hypermobility might be painful as the anatomic borders for movement may be crossed, and inflammation may occur. The body will try to stabilize a hypermobile joint, and spondylosis might occur. In the same breed, [10] found increasingly more spondylosis with increasing age and weight, and a higher prevalence in females than in males. A high degree of heritability has also been documented [10,11,15]. Still, in spite of breeding restrictions since the 1990s, there has been no real reduction in the frequency of spondylosis in Boxers in Norway since then. According to unpublished data compiled by the Norwegian Boxer Club and made available to the authors, an average of 30.3% of the Boxers in Norway born during 2009–2019 had some degree of spondylosis at one year of age. Hence, there is a need to explore further alternative treatments and approaches to reduce the occurrence of the disease.

If the dog has symptoms of backpain, there are several possible treatments. Besides medication with nonsteroidal anti-inflammatory drugs (NSAIDs), Galliprant and Karsivan [18], various food supplements like glucosamine, methylsulphonylmethane (MSM), chondroitin sulphate, and more may have an effect [19,20,21,22,23,24]. Treatments like veterinary chiropractic, osteopathy, acupuncture, physiotherapy, and massage have also shown encouraging results [22,24]. A study on horses [25] has shown an enhanced symmetry and movement of the spine and pelvis after chiropractic treatment, suggesting a similar effect in dogs. It remains to be shown, however, whether enhancing the flexibility and movement of the spine through veterinary chiropractic treatment could also be used to reduce the probability of development of spondylosis. 

We hypothesized that an early development of spondylosis in Boxers may be prevented if segments of the spine with decreased flexibility are detected early and treated with veterinary chiropractic. This was based on the reasoning that the overall flexibility of the spine might normalize; hence, spondylosis might not form, since arthrosis and spondylosis typically form in joints where there is too much movement or irritation. We also hypothesized that the numbers of joints with reduced mobility, which may indicate an unstable and hypermobile back and correlates with the probability of getting spondylosis. To test our hypotheses, puppies from 17 Boxer litters were treated with veterinary chiropractic once a month from eight weeks of age until they were one-year-olds. The status of the treated dogs with respect to spondylosis development was compared with untreated dogs from the same litters at an age of one year, i.e., at the age when Boxers are routinely X-rayed to check for spondylosis in Norway.

## 2. Materials and Methods

The study was performed as a randomized case-control experiment at the veterinary practice owned by the main author. Treatment of the first dogs started in 2009, and the grading of the last X-ray images was completed in 2020. We wanted to include entire litters in the study and to obtain a total sample size of 100 puppies, equally distributed among treated and untreated animals (thus allowing to have full siblings as a control group in each experimental unit). Obtaining puppies for the study was facilitated by breeders connected to the Norwegian Boxer Club, who recruited suitable owners and puppies.

The veterinary chiropractic treatment consisted of checking the mobility of each joint in the back (both the intervertebral joints and the facet joints), neck, and pelvis. The treatment was applied once a month from 8 weeks of age until the dog was one year old and ready to be X-rayed in the spine, ideally at the same time as the rest of the litter. When detecting reduced or no movement in the anatomical direction of the joint, this was corrected with a high velocity and low amplitude thrust as taught at veterinary chiropractic schools approved by the International Veterinary Chiropractic Association or similar organizations. For further details on veterinary chiropractic, see, e.g., [26,27].

**Figure 1:**
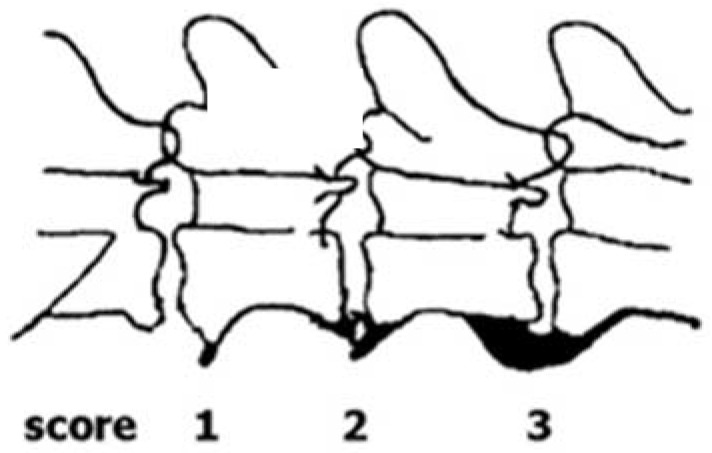
The Norwegian scoring system of enthesophyte development in dogs (reproduced from [10] with permission from *J Small Anim Pract*). A score of 0 (not shown) corresponds to no enthesophyte development.

**Figure 2:**
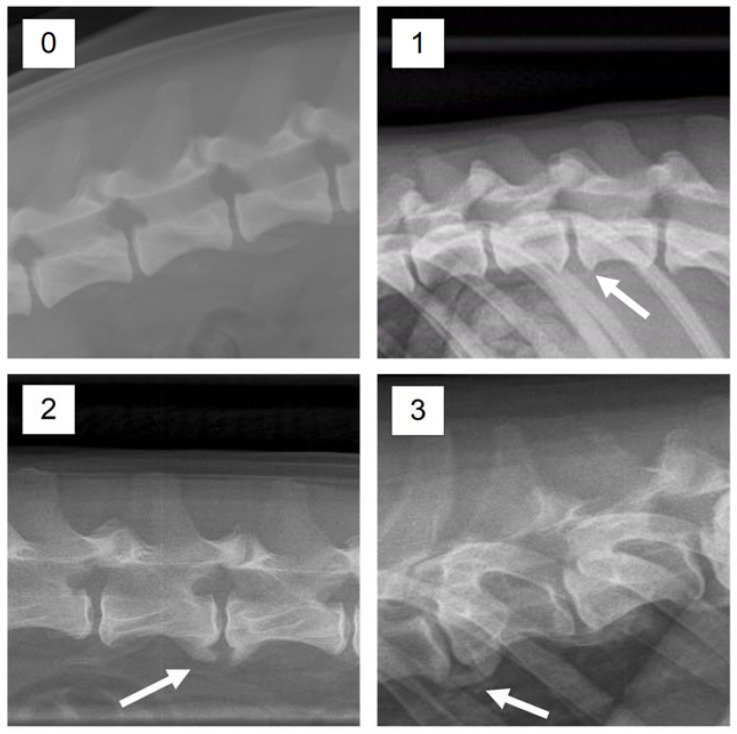
X-ray images with examples of enthesophyte development corresponding to the scores 0–3.

The status with respect to spondylosis formation was assessed from lateral X-ray images of the spine, obtained at an age of ca. one year. In line with current practice in Norway [9], spondylosis was scored manually into one of the four categories 0–3, where the obtained score depends on the size of the ventral arthrosis of the spine (Figure 1 and Figure 2). The scoring considers the spine from T1-S1. A score of 0 corresponds to no visible signs of spondylosis. A dog given a score of 1 has only small bone spurs, while a dog obtaining a score of 2 has more developed bone spurs, but not like a full bridge as is characterizing dogs obtaining a score of 3. It should be kept in mind that the scoring describes the worst finding only, which means that a dog with a score of 1 can have mild spondylosis in one place or along the whole spine. The same goes for a score of 3, which means that a full bone bridge can be present in only one joint, at joints along the whole spine, or something in between. In the statistical analyses we therefore decided to transform the scores obtained from the interpretation of the X-ray images to a binary response variable, with the score values 1–3 treated as occurrence of spondylosis, while a dog who obtained a score of 0 was considered healthy. The scoring was done by trained personnel at the Norwegian Kennel Club (NKK), and the results are available at DogWeb (www.nkk.no). The person that scored the images did not know about the objectives of the study.

The statistical analyses were performed using SAS/STAT^®^ software version 9.4 for Windows. A mixed modeling approach with the procedure GLIMMIX was used to test whether the probability of having spondylosis at one year of age was dependent on the applied chiropractic treatment. Litter was specified as a random effect in the model. Covariates included as fixed effects were sex (male = 1, female = 0) and the spondylosis scoring obtained for the parents (Table 1). Information on the latter was available from the NKK database and was used to derive a categorical variable with three levels (0 = both parents had obtained a score of 0; 1 = only one parent had a score of 1 or higher; 2 = both parents had a score of 1 or higher). The father of litter no. 15 had obtained a score of 0.33 using the Italian scoring system, which was considered equivalent to a score of at least 1 with the Norwegian system. 

An additional model was set up to test if the probability of having spondylosis at one year old correlated with the number of spinal joints with reduced flexibility. Since the total number of chiropractic treatments differed between puppies, the explanatory variable was calculated as the total number of spinal joints with decreased mobility detected for each puppy, divided by the number of times the puppy had been treated. The categorical covariates accounting for sex and spondylosis grading of the parents were included also in this model. 

Although the intention was to give the puppies in the treated group chiropractic treatment at least ten times, this was not achieved in all cases (Table 1). All were treated at least seven times, however, except for one puppy in litter no. 14 which was only treated twice. Excluding this dog from the data did not impact the overall significance of the treatment effect, so it was retained in the data used for the final statistical analysis. 

## 3. Results

Originally, we recruited 19 litters with a total of 110 dogs. However, not all the owners followed up with X-raying their dogs, so in several litters the final sample was less than the number of dogs included in the study initially. We also had to discard two entire litters due to the same reason, since only treated dogs were among the X-rayed ones in those litters. When accounting for an additional puppy from the control group that died before one year of age, the final sample size comprises 87 dogs, 44 of them that were treated with veterinary chiropractic and 43 controls—distributed among 17 litters (Table 1).

**Figure 3:**
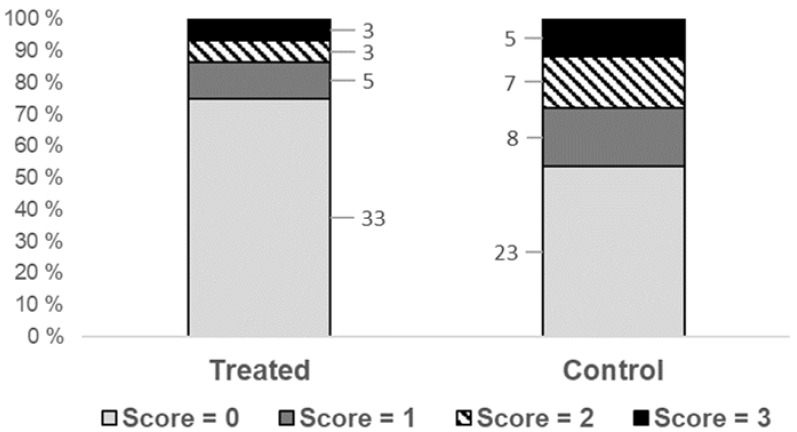
Frequency distribution for the scoring of spondylosis in one-year-old Boxers given veterinary chiropractic treatment (treated) or no treatment (control) as puppies. The number of dogs in each score category is shown next to the bars.

Overall, 35.6% of the dogs that were X-rayed had spondylosis, corresponding to a score between 1 and 3. The corresponding percentages for the dogs given chiropractic treatment and the untreated controls were 25.0 and 46.5 %, respectively (Figure 3). The probability of having spondylosis was significantly lower for the treated group than for the controls (*p* = 0.0478; Table 2). The odds ratio estimate for the treated dogs versus the controls was 0.335, with upper and lower 95% confidence boundaries at 0.114 and 0.989, respectively. Neither the effect of sex nor the effect of spondylosis scoring for the parents were statistically significant, although our data indicated a tendency towards a lower occurrence when both parents are healthy, compared to when both have spondylosis (*p* = 0.1144; Table 2).

The average number of spinal joints with decreased mobility found per chiropractic treatment was 3.9, ranging between 1.3 and 6.1 depending on the puppy. We did not find any significant effect of this variable upon the occurrence of spondylosis in the treated group of dogs, however (*p* = 0.1445; Table 3). The dataset used in the statistical analyses is provided as Supplementary Material in Table S1.

## 4. Discussion

We wanted to see if it was possible to prevent the early occurrence of spondylosis in Boxers by optimizing the movement in the spine with veterinary chiropractic from the age of eight weeks until they are X-rayed at one year of age. Our results showed that the odds of obtaining a score of 1 or higher with the Norwegian grading system was significantly lower in the treated dogs than in the control group, hence confirming our first hypothesis. To our knowledge, no studies have previously examined whether chiropractic treatment can effectively counteract the development of spondylosis when used as a precautionary measure prior to the development of symptoms. A statistically significant correlation between the occurrence of spondylosis and the average number of spinal joints with decreased mobility in the treated dogs was not found, however, which is contrary to our second hypothesis. This could still be a possible explanation as to why the chiropractic treatment counteracted the early development of spondylosis. To better clarify any such causal relationship, further studies with larger data material would probably be needed.

The overall frequency of spondylosis for the dogs in this study was 35.6 per cent, which is only slightly higher than the average frequency for Norwegian Boxers born between 2009 and 2019 (30.3%, *n* = 1511, statistics obtained from the Norwegian Boxer Club). While the frequency of occurrence for the treated dogs (25.0%) is below the recorded 11-year average, the average for the controls (45.6%) is close to the upper range of the observed variation. The result for both groups is within the variation range (25-51%) recorded for Norwegian Boxers born in the different years during this 11-year period, however.

While the chiropractic treatment was our main factor of interest, we also opted to account for: (1) the spondylosis scoring of the parents and (2) sex, by including these factors as covariates in the mixed models. Given the modest sample size of this study, we did not expect to be able to infer much about the effect of these factors *per se*. However, previous studies have shown a higher prevalence in females than in males [10,15] as well as a clear hereditability effect [9,10] on spondylosis occurrence. We therefore wanted to account for the likely confounding effect of these factors in the statistical analyses, since our data was partly unbalanced both with respect to both the total number and the proportion of males and females in the different litters. It may be noted that although the effects of these two covariates were not statistically significant, the direction of the respective parameter estimates are in line with the findings in the above-mentioned studies [9,10,15]. The experimental design with treatments assigned to dogs of the same litter also made it possible to account for the influence of other potentially influencing conditions or “noise” specific to each litter, such as differences in feeding, physical exercising, use of harnesses or other types of training. 

To test our hypotheses, we combined the four-level spondylosis scoring to a binary (0, 1) response variable, i.e., considering each dog to either have spondylosis or not. When running the statistical analyses, we also considered using the original scores as an ordinal variable, i.e., implicitly considering the scoring to reflect a severity gradient. However, such an assumption is, in our opinion, not straightforward. This is because the highest score will be obtained in any case if a full bony bridge is observed only on a single place along the spine, i.e., even if the rest of the spine has no symptoms. On the other hand, a dog can have bone spurs on several ligaments along most of the spine, yet it will obtain a score of 1 if no higher order enthesophytes are observed along the spine. Considering these limitations, we are of the opinion that the approach with treating the response as a binary variable was the most appropriate choice. We also performed the same analyses using the original scores as an ordinal response variable, however. This reduced the *p*-value of the treatment effect from 0.0478 to 0.0652. The difference is largely due to the reduction in the number of degrees of freedom in the denominator for the testing of the fixed effects (i.e., a reduction from 69 to 67 when choosing the latter approach).

While the current X-ray screening of Boxers in Norway also yields information on the site of occurrence of spondylosis along the spine, this was not recorded in the screening program during the first years of the duration of our study. Obviously, such information would have been valuable as a supplement to the scoring results obtained from the X-ray image interpretation. Moreover, it is indeed very likely that MR images could have provided even more detailed information. X-ray screening is, however, the only method that is currently available to dog owners given the high cost of MR screening. We hence opted to use the data which was made available to us from the Norwegian Kennel Club through the routinely conducted X-ray scanning of Boxers in Norway.

In conclusion, our results were encouraging, providing novel evidence that veterinary chiropractic treatment may be successfully used to reduce the probability of early development of spondylosis in young Boxers. Chiropractic treatment is not currently used as a routine treatment of spondylosis due to a lack of evidence so far that the treatment can have an effect. The goal of our study was to test if it is possible to reduce the early occurrence of spondylosis as such. Hence, whether such treatment will be effective in reducing spondylosis development at later life stages still remains an open question. It should also be noted that the total number of dogs was rather low in this study, which is also reflected in the wide confidence interval for the odds ratio. Clearly, a follow-up study with larger data material to corroborate our findings would be highly valuable. 

## Figures and Tables

**Table 1:** Summary of litters included in the study. The number of puppies that were X-rayed make up the final sample included in the statistical analyses. Details on the variables recorded for the individual dogs included in the final sample, including their spondylosis score, are available as Supplementary Material in Table S1.

Litter No.	No. of Dogs in Total		Final Sample (X-rayed)		No. of Treatments	Parent Score (Male/Female)
	Treated (Male/Female)	Control (Male/Female)	Treated (male/female)	Control (male/female)		
1	3 (1/2)	3 (1/2)	2 (0/2)	2 (0/2)	10	0/0
2	5 (2/3)	4 (4/0)	5 (2/3)	4 (4/0)	10–11	2/0
3	3 (3/0)	4 (1/3)	3 (3/0)	4 (1/3)	7–11	0/3
4	3 (1/2)	4 (2/2)	3 (1/2)	4 (2/2)	7–8	0/0
5	3 (1/2)	3 (0/3)	3 (1/2)	3 (0/3)	10	1/0
6	3 (2/1)	1 (1/0)	3 (2/1)	1 (1/0)	7–8	0/3
7	3 (2/1)	4 (1/2)	3 (2/1)	3 (1/2)	8	1/0
8	4 (3/1)	3 (3/0)	4 (3/1)	2 (2/0)	9–11	1/0
9	2 (1/1)	2 (1/1)	2 (1/1)	2 (1/1)	9–10	0/0
10	2 (1/1)	4 (3/1)	2 (1/1)	4 (3/1)	7–10	0/1
11	1 (0/1)	2 (1/1)	1 (0/1)	1 (0/1)	8	0/0
12	2 (1/1)	3 (0/3)	1 (0/1)	2 (0/2)	10	2/0
13	2 (1/1)	2 (1/1)	2 (1/1)	2 (1/1)	2–7	0/0
14	2 (0/2)	5 (3/2)	1 (0/1)	1 (1/0)	11	0/0
15	4 (1/3)	2 (1/1)	4 (1/3)	2 (1/1)	11	0/0,33^1)^
16	3 (1/2)	3 (0/3)	3 (1/2)	2 (0/2)	7–11	0/0
17	3 (0/3)	6 (2/4)	2 (0/2)	4 (1/3)	9–10	2/1
Total	48 (21/27)	55 (25/30)	44 (19/25)	43 (19/24)		

**Table 2:** Parameter values for the model testing for spondylosis occurrence in one-year-old Boxers given repeated chiropractic treatment as puppies or no treatment. The default settings (parameter estimate = 0) for the categorical fixed effect parameters are: chiropractic treatment = yes; sex = female; and both parents having a score ≥ 1 according to the Norwegian scoring system.

Parameter	Estimate	SE	DF	*t*-Value	*p*-Value
Intercept	0.1485	1.1221	14	0.13	0.8966
Chiropractic treatment = no	1.0924	0.5420	69	2.02	0.0478
Sex = male	−0.5858	0.5876	69	−1.00	0.3233
Parent with spondylosis = none	−2.1213	1.3266	69	−1.60	0.1144
Parent with spondylosis = one	−1.0122	1.2183	69	−0.83	0.4089
σ^2^ litter	1.1798	0.9677			

**Table 3:** Parameter values for model testing for the effect of the number of spinal joints with decreased mobility on spondylosis occurrence in one-year-old Boxers given repeated chiropractic treatment as puppies. The default settings (parameter estimate = 0) for the categorical fixed effect parameters are: sex = female and both parents having a score ≥ 1 according to the Norwegian scoring system.

Parameter	Estimate	SE	DF	*t*-Value	*p*-Value
Intercept	−1.8965	1.6102	14	−1.18	0.2585
Spinal joint mobility ^(1)^	0.5449	0.3621	26	1.50	0.1445
Sex = male	−0.7042	0.8479	26	−0.83	0.4138
Parent with spondylosis = none	−1.3240	1.0951	26	−1.21	0.2375
Parent with spondylosis = one	−1.3780	1.0970	26	−1.26	0.2202
σ^2^ litter	0	−			

^(1)^ Total no. of spinal joints with decreased mobility divided by no. of chiropractic treatments.

## Data Availability

The full dataset used in the statistical analyses is provided as supplementary material in Table S1.

## Supplementary Materials

The following are available online at https://www.mdpi.com/article/10.3390/vetsci8090199/s1, Table S1: Scoring results and additional data used in the statistical analyses of spondylosis in young Boxers.
