# Supplementary material for: Veterinary Chiropractic Treatment as a Measure to Prevent the Occurrence of Spondylosis in Boxers
Source: Vet Sci. 2021 Sep 17;8(9):199. doi: 10.3390/vetsci8090199 (PMC8473340; doi:10.3390/vetsci8090199)
Supplement: Supplementary file 1 [file vetsci-08-00199-s001.zip › vetsci-1277109-supplementary.pdf]

Table S1. Dataset used in the final statistical analyses. Information about the number of dogs excluded from each litter and on the spondylosis score of the parents of each litter and may be obtained from Table 1.

Litter #	Dog # within Litter	Treatment Group	No. of Times Treated	Sex	No. of Joints with Decreased Mobility	Spondylosis Score
1	1	Treated	10	Female	49	0
	2	Treated	10	Female	29	0
	3	Control	0	Female	-	0
	4	Control	0	Female	-	0
2	1	Treated	11	Female	43	0
	2	Treated	11	Male	43	0
	3	Treated	10	Female	26	0
	4	Treated	11	Female	40	0
	5	Treated	10	Male	40	0
	6	Control	0	Male	-	0
	7	Control	0	Male	-	0
	8	Control	0	Male	-	0
	9	Control	0	Male	-	0
3	1	Treated	11	Male	58	0
	2	Treated	7	Male	38	0
	3	Treated	11	Male	62	1
	4	Control	0	Male	-	0
	5	Control	0	Female	-	1
	6	Control	0	Female	-	2
	7	Control	0	Female	-	1
4	1	Treated	8	Male	24	0
	2	Treated	8	Female	34	2
	3	Treated	7	Female	22	0
	4	Control	0	Male	-	1
	5	Control	0	Male	-	3
	6	Control	0	Female	-	1
	7	Control	0	Female	-	0
5	1	Treated	10	Female	61	0
	2	Treated	10	Female	22	0
	3	Treated	10	Male	51	0
	4	Control	0	Female	-	0
	5	Control	0	Female	-	0
	6	Control	0	Female	-	1
6	1	Treated	8	Male	46	3
	2	Treated	7	Female	27	3
	3	Treated	8	Male	11	0
	4	Control	0	Male	-	2
7	1	Treated	8	Male	40	0
	2	Treated	8	Female	42	0
	3	Treated	8	Male	10	0
	4	Control	0	Male	-	0
	5	Control	0	Female	-	3
	6	Control	0	Female	-	1

Litter #	Dog # within Litter	Treatment Group	No. of Times Treated	Sex	No. of Joints with Decreased Mobility	Spondylosis Score
8	1	Treated	11	Male	39	0
	2	Treated	11	Male	39	0
	3	Treated	9	Female	39	2
	4	Treated	10	Male	23	0
	5	Control	0	Male	-	0
	6	Control	0	Male	-	2
9	1	Treated	9	Female	49	1
	2	Treated	10	Male	49	0
	3	Control	0	Female	-	0
	4	Control	0	Male	-	0
10	1	Treated	10	Female	33	2
	2	Treated	7	Male	25	0
	3	Control	0	Male	-	2
	4	Control	0	Male	-	3
	5	Control	0	Male	-	2
	6	Control	0	Female	-	0
11	1	Treated	8	Female	21	0
	2	Control	0	Female	-	0
12	1	Treated	9	Female	45	0
	2	Control	0	Female	-	2
	3	Control	0	Female	-	0
13	1	Treated	2	Female	9	0
	2	Treated	7	Male	16	0
	3	Control	0	Female	-	0
	4	Control	0	Male	-	1
14	1	Treated	11	Female	39	0
	2	Control	0	Male	-	0
15	1	Treated	11	Female	48	0
	2	Treated	11	Female	37	0
	3	Treated	11	Female	38	0
	4	Treated	11	Male	21	1
	5	Control	0	Male	-	0
	6	Control	0	Female	-	0
16	1	Treated	11	Male	21	0
	2	Treated	11	Female	29	0
	3	Treated	7	Female	26	1
	4	Control	0	Female	-	0
	5	Control	0	Female	-	0
17	1	Treated	9	Female	40	1
	2	Treated	9	Female	38	3
	3	Control	0	Female	-	3
	4	Control	0	Female	-	3
	5	Control	0	Female	-	1
	6	Control	0	Male	-	2
